# Immunotherapy in extensive small cell lung cancer

**DOI:** 10.1186/s40164-019-0129-x

**Published:** 2019-02-04

**Authors:** Vaibhav Verma, Geeti Sharma, Abhijai Singh

**Affiliations:** 1 Mon Health Medical Center, Morgantown, WV USA; 2 Morgantown, WV USA; 3 Steward Medical Group, Trumbull Medical Center, Youngstown, OH USA

## Abstract

Small cell lung cancer which constitutes about 15% of lung cancers is pathobiologically and clinically distinct from non small cell cancer. Histologically it is characterized by small cells with scant cytoplasm, absent or inconspicuous nucleoli, extensive necrosis, and expresses neuroendocrine markers. It is on a spectrum of neuroendocrine cancer that extend from typical carcinoids to large cell to small cell cancer. Clinically it behaves in a more malignant fashion with a rapid doubling time, early metastasis. They respond rapidly to cytotoxic treatment however tend to develop resistance soon. Immunotherapy with checkpoint inhibitors take advantage of PD 1 ligand-receptor axis between the tumor and T cells or CTLA4 on T cells which when engaged lead to inhibition of T cells. This inhibition helps tumors to evade immune surveillance. Checkpoint inhibitors break this axis by either binding to PD 1 ligands or PD 1 to CTLA4, thereby preventing tumors to evade the immune systems. This has led to remarkable responses in tumors. The immune related adverse effects can be severe however are experienced at much lower rates as compared to cytotoxic chemotherapy. Recently, CheckMate 032 has shown impressive response rates with Nivolumab and Nivolumab/Ipilimumab in relapsed small cell cancer. IMpower 133, a phase 3 trial showed that addition of Atezolizumab to Carbo/Etoposide led to a significant survival benefit in treatment naive extensive small cell cancer. This review will summarize recent developments and ongoing studies of immune therapy in extensive small cell cancer in addition to a brief summary of immune therapy landscape of Non small cell lung cancer. Investigational approaches to immune therapy have also been delineated.

## Introduction

A surgeon by the name William Coley reported in 1893 that repeated inoculations of killed bacteria into sarcomas led to their shrinkage, possibly laying the foundation of immune therapy in Oncology [1]. Medical Oncology has come a long way since then although immune therapy had sort of been on the back burner until in the last few years where it has touched almost all forms of cancer and changed the landscape of cancer treatment.

An efficient cytotoxic immune response against a tumor requires a complex interaction at the immune synapse which consists of various stimulatory and inhibitory receptors. PD L1 on tumors and PD 1 on T cells are one such type of inhibitor receptors that form an axis, which when engaged leads to inhibition of T cells, thereby allowing tumors to evade immune surveillance [2, 3].

Small cell cancers differ from non small cell cancer by a rapid doubling time, early metastasis and frequent brain mets. They constitute about 15% of all lung cancer diagnosis and a SEER analysis showed a decrease in proportion of small cell cancers over last few decades [4]. A diagnosis of small cell cancer portends a poor prognosis—20 to 40% of limited stage and less than 5% of extensive stage patients survive 2 years [5]. Small cell cancers are almost exclusively diagnosed in smokers [6].

While non small cell cancer has had other developments—in addition to immunotherapy—in past years whereby actionable driver mutations were discovered and led to marked improvements in outcomes, small cell cancer therapy treatment landscape had remained absolutely unchanged for past 2 decades. This changed recently as immune therapy has in the past few months, for the first time in last few decades showed promise in small cell cancer. In this article we have reviewed recent developments in small cell cancer that are practice changing, ongoing trials and investigational approaches.

### Immune therapy in relapsed extensive small cell cancer

CheckMate 032 is a phase 1/2 multi center trial studying Nivolumab or Nivolumab combined with Ipilimumab in advanced/metastatic solid tumors [7]. The non randomized small cell lung cancer (SCLC) cohort of this trial, which consisted of patients with progressive SCLC after platinum therapy, was presented in 2016 and showed an overall response rate (ORR) of 10% with Nivolumab and 23% with Nivolumab 1 mg/kg and Ipilimumab 3 mg/kg, with grade 3–4 adverse effects of 14% and 33% in Nivolumab and Nivolumab plus Ipilimumab respectively.

The promising results prompted a randomized expansion cohort where 247 patients were randomized to Nivolumab and Nivolumab 1 mg/kg plus Ipilimumab 3 mg/kg [8]. Overall response rate of 12% and 21% were seen in Nivolumab and Nivolumab plus Ipilimumab arms respectively. Responses were observed regardless of platinum sensitivity, PD L1 status or line of therapy. 3 months OS was similar at 64% and 65% for Nivolumab and Nivolumab/Ipilimumab respectively.

These results were the basis for FDA (Food and Drug Administration) approval of Nivolumab although immune therapy was not compared to the standard of care for relapsed SCLC, i.e. chemotherapy. Historically, Topotecan has been associated with ORR of around 20% and grade 3–4 neutropenia and thrombocytopenia of 30–50% [9]. Topotecan has also been associated with overall survival (OS) benefit compared to best supportive care (BSC) [10].

### Immune therapy in chemotherapy naive extensive small cell cancer

In a randomized phase 3 trial studying treatment naive extensive small cell cancer, 403 patients were assigned to Carboplatin plus Etoposide with either Atezolizumab (for 4 cycles) or placebo followed by either Atezolizumab or placebo maintenance till progression/intolerance/no more benefit [11]. PD-L1 testing was not performed owing to expected high rate of inadequate sample types (e.g., fine needle aspirates), low prevalence of PD-L1 expression on tumor cells, and lack of association between response and PD-L1 expression in phase 1 trial of Atezolizumab in extensive small cell lung cancer. At a median follow up of 13.9 months the median OS for Atezolizumab plus chemo vs. placebo plus chemo arm was 12.3 vs. 10.3 months (hazard ratio—HR-for death 0.7, 95% confidence interval (CI) 0.54 to 0.91). Median progression free survival (PFS) for Atezolizumab plus chemo vs. placebo plus chemo arm was 5.2 vs. 4.3 months with hazard ratio of 0.77, CI 0.62 to 0.96. Grade 3–4 adverse effects were similar—58% in each group.

### On going trials in small cell lung cancer

CheckMate 331, an ongoing phase 3 randomized trial comparing Nivolumab with chemo-Topotecan in relapsed SCLC should be helpful in answering questions about appropriate management of a relapsed SCLC in currently era of immune therapy [12].

CheckMate 451 is an ongoing phase 3 trial assessing Nivolumab and Nivolumab/Ipilimumab vs. placebo as maintenance in extensive small cell cancer after first line platinum based doublet chemotherapy [13].

Keytruda was studied in KeyNote 158 which is a phase 2 basket study of 11 cancer types [14]. PD L1 positive was defined as PD L1 combined positive score ≥ 1. ORR of 18% was observed overall with 35% in PD-L1 positive tumors and 6% in PD-L1 negative tumors. Median OS was 9 months overall, 14.6 months in PD-L1 positive and 7.7 months in PD-L1 negative tumors. Keytruda is not yet approved for small cell cancer.

KeyNote-028 is an ongoing phase 1b trial studying Keytruda for patients with pretreated, extensive PD-L1 + SCLC. PD-L1 positive was defined as > 1% PD-L1 expression. Of the initial 16 patients evaluated, 53% developed treatment related adverse effects with only 1/16 developing grade 3 toxicity. Twenty-five percent of the patients had a partial response, and 7% had stable disease with 37% of patients with progressive disease [15]. The relevant trials have been summarized in table 1.

**Table 1:** Selected small cell lung cancer trials

Name of trial	Phase	Therapy	mOS	mPFS	ORR (%)	Ref
CheckMate 032 (relapsed extensive stage)	1/2	Nivolumab 3 mg/kg	NR	NR	10	[7]
		Nivolumab 1 mg/kg + Ipilimumab 1 mg/kg	NR	NR	33	
		Nivolumab 1 mg/kg + Ipilimumab 3 mg/kg	NR	NR	23	
		Nivolumab 3 mg/kg + Ipilimumab 1 mg/kg	NR	NR	19	
IMpower 133 (treatment naive extensive stage)	3	Carboplatin/Etoposide 4 cycles	10	4.3		[11]
		Carboplatin/Etoposide/Atezolizumab 4 cycles—Atezolizumab maintenance	12	5.2		

Name of trial	Ongoing trials					
	Phase	Therapy	Primary endpoints	Secondary endpoints	Ref	
CheckMate 331 (relapsed)	3	Nivolumab	OS	PFS, ORR	[12]	
		Topotecan				
CheckMate 451 (maintenance after response to platinum doublet	3	Nivolumab	OS	PFS	[13]	
		Nivolumab + Ipilimumab				
		Placebo				
KeyNote 158 (relapsed/refractory)	2	Keytruda	ORR	OS, PFS, DOR	[14]	
KeyNote 028 (extensive stage)	1	Keytruda	ORR	OS, PFS, DOR	[15]	

*mOS* median overall survival, *mPFS* median progression free survival, *ORR* objective response rate, *Ref* reference, *NR* not reached, *OS* overall survival, *PFS* progression free survival, *DOR* duration of response

### Role of tumor mutation burden (TMB) in small cell cancer

The lack of correlation of response in Small cell lung cancer to PD-L1 expression requires exploration of other biomarkers for predicting response. A detailed biomarker analysis of patients enrolled in above mentioned CheckMate 032 trial was done by Hellmann et al. [16]. 27% of patients analyzed were found to have high tumor mutation burden—TMB high—as determined by whole exome sequencing. Among TMB-high patients 1 year overall survival was 35% for patients treated with Nivolumab and 62% for patients treated with nivolumab and Ipilimumab. The 1 year overall survival was 20–26% in low/medium TMB in patients who received Nivolumab + Ipilimumab. In contrast to TMB, PD-L1 was positive in 12% of the patients and was not predictive of response.

### Immune therapy landscape in non small cell cancer

Immune therapy is being used for Non small cell cancer now for a few years, although in recent months there have been developments that are worth mentioning. In 2015 CheckMate 017 was published [17]. 272 patients with advanced squamous cell lung cancer who had disease progression on or after first line chemotherapy, were assigned to Nivolumab vs. Docetaxel. Median overall survival was 9.2 vs. 6 months in Nivolumab vs. Docetaxel arm respectively. Hazard ratio for death was 0.59; 95% CI 0.44 to 0.79, p < 0.001. Treatment related adverse events of grade 3 or 4 were reported in 7% of the patients in Nivolumab group vs. 55% of the patient in Docetaxel group. About half the patients had PD L 1 expression of at least 1%, however the PD L1 expression did not effect survival. Around same time CheckMate 057 was published where 582 patients with advanced non squamous non small cell lung cancer who progressed on or after platinum chemotherapy were randomized to Nivolumab vs. Docetaxel. Median OS in Nivolumab group was 12.2 months vs. 9.4 months in Docetaxel group with HR for death of 0.73; 95% CI 0.59 to 0.89, p − 0.002 [18].

In a phase III trial with 1225 patient with PD L1 unselected, advanced non small cell lung cancer with disease progression on or after platinum doublet chemotherapy, Atezolizumab versus Docetaxel improved median OS (13.8 months versus 9.6 months with hazard ratio for death of 0.73; 95% CI 0.62–0.87). Grade ≥ 3 treatment related toxicities were 15% vs. 43% in Atezolizumab vs. Doetaxel [19]. PD-L1 positive was defined as ≥ 1% expression of PD-L1 on tumor cells or tumor infiltrating immune cells.

KeyNote-010 was published in 2016 which randomized 1034 patients with advanced Non small cell cancer patients previously treated with platinum chemotherapy and had at least 1% PDL1 expression, to Pembrolizumab 2 mg/kg (low dose), 10 mg/kg (high dose) or Docetaxel. Median overall survival was 10.4 months vs. 8.5 months in low dose Pembrolizumab vs. Docetaxel group with hazard ratio for death of 0.71; 95% CI 0.58–0.88). Patients with PDL 1 expression > 50% derived the greatest survival benefit [20].

The above mentioned trials established the role of immune therapy in advanced non small cell cancer with prior treatment with platinum chemotherapy. Immune therapy was then tested in first line setting in advanced non small cell cancer. KeyNote-024 was published in 2016 in which 305 treatment naive patients with advanced non small cell cancer with at least 50% PD L1 expression were randomized to Pembrolizumab mono therapy or standard platinum doublet chemotherapy [21]. Median overall survival in Pembrolizumab mono therapy vs. chemotherapy was an impressive 30 months versus 14.2 months with hazard ratio for death of 0.63; 95% CI 0.47–0.86). Grade ≥ 3 treatment related adverse effects were 27% vs. 53% in Pembrolizumab vs. chemotherapy group. KeyNote 189 published in 2018 randomized 616 patients with treatment naive advanced Non Squamous Non Small Cell Cancer, unselected for PD L1 expression to receive either Cisplatin/Carboplatin and Pemetrexed with or without Pembrolizumab [22]. 12 month overall survival in chemo plus Pembrolizumab vs. chemo only arm was 69% vs. 49% with hazard ratio for death of 0.49; 95% CI 0.38–0.64. Overall survival benefits were seen in all PD Ll groups. PD-L1 was defined as ≥ 1% expression of PD-L1 on tumor cells. Grade ≥ 3 treatment related adverse effects were seen in 67% of patients in chemo plus Pembrolizumab vs. in 66% of patients in chemo only group. KeyNote 407 published in 2018 randomized 559 patients with treatment naive advanced squamous non small cell cancer, unselected for PD L1 expression to receive either Carboplatin and paclitaxel/nabpaclitaxel with or without Pembrolizumab [23]. Median overall survival was 15.9 months versus 11.3 months in chemo plus Pembrolizumab versus chemo arm with hazard ratio for death of 0.64; 95% CI 0.49–0.85. Grade ≥ 3 treatment related adverse effects were seen in 70% vs. 68% of patients in chemo plus Pembrolizumab versus chemo only arm. PD-L1 positive was defined as ≥ 1%.

### Problem with low response rate and adequate selection of patients

It can be seen from above mentioned trials that the response rates of immune therapy continues to be low. A host of factors could account for that, one of them being inability to adequately predict patients who would benefit from immune therapy. In general the average specificity and specificity of PD L1 IHC (immuno histochemistry) assays with their respective cut off, for response is 58% and 72% respectively [24]. This implies that 42% of patient who are not likely to respond to immune therapy would be treated with immune therapy due to them being labeled as PD L1 positive and on the other hand 28% of patients who would not be considered for immune therapy due to being labelled as P-L1 negative would have benefitted for immune therapy. Hence the need for better predictors for response to Immune therapy. Various factors in the tumor microenvironment as well tumor intrinsic features other than just PDL L1 expression can effect the tumor response to Immune therapy.

#### Tumor microenvironment related factors

Due to intra tumor heterogeneity and changes in PDL1 expression over time along with treatment and/or progression, a small biopsy specimen at one point of time would mis represent the PD L 1 expression of the disease as a whole [25, 26]. Also, up regulation of PD-L1 as measured by IHC assays is caused by intracellular oncogenic variations as well as exposure to cytokines like IFN-gamma from tumor infiltrating lymphocytes. However, only immunity induced up regulation is more predictive of tumors response to PDL1/PD1 inhibition [27]. Due to alterations in the microenvironment by other treatments, the prediction of benefit for PDL1/PDI inhibitor when used in combination regimens is difficult [28].

Tumor infiltrating lymphocytes (TIL) are an important influence on the tumor microenvironment. Presence of high density of TIL has been shown to be predictive of good response to immune therapy [29, 30]. IFN-gamma expression (secreted by TIL) is generally believed to predict a favorable response to anti PDL1/PD1 therapy [31].

#### Tumor intrinsic features related factors

Tumor mutation burden which reflects accumulated mutations, results in increased immunogenicity and thus creates a microenvironment favorable for PD L1/PD1 inhibition [32, 33]. Mismatch repair protein deficiency or Micro satellite instability leads to accumulation of mutations as well production of neoantigen and predicts a favorable response to PDL1/PD1 therapy [34, 35]. Presence of certain driver mutations also affect PDL-1/PD1 axis. Mutated EGFR (epidermal growth factor receptor) up regulates PD-L1 but impedes TIL at the same time and in general patients with mutated EGFR tend to have a poor response to PD-L1/PD1 therapy when compared to wild type EGFR [36]. Patients with mutated KRAS (kirsten rat sarcoma) are more likely to belong to PDL 1 subtype [37]. ALK (anaplastic lymphoma kinase) rearrangement has been associated with low PD L1 expression [38].

### Role of neoantigen and tumor mutation burden

The role of neoantigen generated by tumor somatic mutation in contributing to tumor microenvironment immunogenicity and induction of anti tumor response is well established [39]. According to the cancer-immunity cycle theory, mutation derived neoantigen released by cancer cell is captured and presented by antigen presentation cell (APC) which induces priming and activation of neo-antigen specific T cells, which then infiltrate to the tumor and kill the tumor cells [40]. Therefore neoantigen is presumed to be biomarker for immune therapy as well as a complimentary target for treatment along with immune-therapy.

#### Tumor mutation burden

Since somatic tumor mutations give rise to neoantigen production, TMB is considered to be a surrogate marker for neoantigen burden and biomarker for anti PDL-1/PD1 therapy [33]. In spite of seemingly being a good surrogate marker, some patients with high TMB respond poorly to checkpoint inhibition. There could be a couple of plausible explanations. Both clonal and subclonal mutation account for the total measured TMB. However high clonal mutation rate predicts a favorable response to PD-L1/PD1 inhibition whereas high subclonal mutation rate predicts a poor response to PD-L1/PD1 inhibition [41, 42]. Single site biopsy might overestimate clonal mutation rate. The other reason possibly is that the TMB quantifies the amount of mutation however it is not the mutation but the neoantigen that effects the tumor immune response [43]. Not all mutations get to produce neoantigens. This process involves a complex process of the mutated protein being processed and bound to MHC (major histocompatibility complex) and presented by antigen presenting cells. Hence the need for being able to predict neoantigen.

#### Prediction of neoantigen and it’s clinical application

Neoantigens can be predicted through algorithms which consists of three parts: (1) identification of mutated portions with the help of next generation sequencing, public databases of genes; (2) identification of MHC typing with the help of sequencing data and public data bases; (3) prediction of binding affinity between MHC and neopeptides [44, 45].

Neoantigen vaccines prepared with the method mentioned above have been tested and shown to have a potent anti tumor response [46]. Some failures to neoantigen vaccines are attributed to immune inhibitory tumor microenvironment—partly contributed by up regulation of PD-L1 due to the vaccine’s effects—which can be overcome by combining neoantigen vaccine with immune checkpoint inhibitor therapy [47, 48].

Neoantigen vaccines could also help overcome resistance to checkpoint inhibition—due to variation of neoantigen repertoire—since immune stimulating component of vaccine could be manipulated depending on dynamic variation of neoantigen spectrum during tumor evolution [49].

Another application of neoantigen prediction could be adoptive T cell transfer. In preclinical trials adoptive T cell transfer targeting tumor specific mutations showed potent anti tumor activity [50–52].

Treatment with radiotherapy and oncolytic virus releases neoantigens from cancer cell by causing death of cancer cells, which might have otherwise remained hidden from immune surveillance [53]. This has the potential to alter the immune surveillance status [53]. Combining radiotherapy or oncolytic virus with immune checkpoint inhibition can take advantage of the altered immune status by increasing anti tumor response [54–56].

### Investigational approaches in Immune therapy

A number of different approaches are being investigated to be able to harness the immune system to treat cancer. They can be broadly classified as checkpoint inhibitors other than PD-L1/PD1 inhibition, CTLA-4 antibodies, agonism of costimulatory receptors, manipulating T cells, Oncolytic viruses, therapies directed at other cell types and vaccines. These are all either being studied in pre clinical animal models or are in early stages of clinical development.

#### Checkpoint inhibitors targets other than PD-L1/PD1 and CTLA-4

Various targets have been identified but all are in early stages of clinical development.

CD47—CD 47 when expressed on tumors prevents them from phagocytosis by macrophages and has been shown to be a potential target for anticancer treatment [57].

BTLA—B and T cell lymphocyte attenuator is a ligand of herpes virus entry mediator (HEVM) whose interaction leads to decreased production of cytokines, cell proliferation by CD4+ T cells and has shown anti tumor response [58].

VISTA—VISTA (V-domain Ig suppressor of T cell activation) shares homology with PD-L1 and is a negative checkpoint ligand and VISTA blockade has demonstrated tumor response [59].

LAG3—Lymphocyte activation gene 3 (LAG3) expressed on immune cells, when bound by major histocompatibility complex (MHC) class II, hampers T cell differentiation and proliferation. Dual PD-1 and LAG3 blockade has shown anti tumor activity [60].

TIM-3—T cell immunoglobulin and mucin domain 3, expressed on immune cells when binds to galectin-9 on tumors causes T-helper-1 cells’ death [61]. Combined blocked of TIM-3 blockade with either CTLA-4 or PD-1, has shown anti tumor activity [62].

#### Agonism of costimulatory receptors

41-BB (CD137)—this is present on T cells and on stimulation by it’s natural ligand—4-1BBL or agonist antibodies, it promotes activity of T cells and has shown anti tumor activity [63].

CD 40—This is a co stimulatory molecule expressed on antigen presenting cells (APC). When it binds to it’s ligand-CD 40L-present on T cells, it leads to activation of a APCs and has shown to demonstrate anti tumor response [64].

ICOS-I—Inducible T cell co-stimulator (ICOS) is expressed on activated T cells. ICOS engagement with it’s ligand has shown anti tumor response when used in combination with CTLA-4 blockade [65].

OX 40 (CD134)—OX40 is expressed on activated T cells. Engagement of OX 40 with it’s ligand OX 40L or an antibody agonist provides a strong stimulatory effect on effector T cells and has shown anti tumor response [66].

GITR (CD357)—Glucocorticoid-induced tumor necrosis factor like receptor (GITR) is expressed on activated T cells and it’s stimulation by anti-GITR antibody DTA-1 has shown to impair regulatory T cells (Tregs) function and improve anti tumor activity of cytotoxic T cells [67].

#### Manipulating T cells

##### Chimeric antigen receptors

Chimeric antigen receptors (CAR) T cells are patients’ own T cells which are genetically modified to express antigen binding domain from a B cell receptor. Recognition of a specific cell surface antigen causes T cell activation. CAR T cells have been most extensively been studies in hematologic malignancies however early stage studies in solid malignancies are ongoing [68, 69].

##### Ex-vivo expansion of tumor infiltrating lymphocytes (TILs)

TILs are extracted from freshly resected tumor tissue and expanded by co-culturing with IL-2. Patient are then given non myeloablative chemotherapy to deplete Treg cells before infusing ex vivo expanded TILs [70]. This approach has shown promising anti tumor response [71].

##### Bispecific T cell engagers

Bispecific T cell engagers (BiTEs) consist of a protein fragment, one end of which recognizes CD3 on T cells and the other end recognizes the target antigen and this causes a T cell activation against a specific tumor antigen in an MHC-subtype independent manner. Blinatumomab, a BiTE has shown activity in relapsed/refractory acute lymphoblastic leukemia and has been approved by FDA for that indication [72].

#### Oncolytic viruses

Genetically engineered viruses can preferentially infect cancer cells over normal cells and promote presentation of tumor associated antigens. This effects the tumor immune microenvironment by increasing T cell infiltration, IFN gamma signaling and up regulating PD-L1 [73]. Combining oncolytic virus with check point inhibitors have shown synergistic anti tumor response [74].

#### Natural killer cells

Natural killer cells infiltration in tumors are associated with good prognosis [75]. They express killer immunoglobulin-like receptor (KIR). These can transduce inhibitory as well as stimulatory signals to the NK cells [76]. Blockade of inhibitory KIR by anti KIR antibodies has shown anti tumor response [76, 77].

## Conclusion

The recent trials outlined above, studying immune therapy in small cell cancer have set new precedents in the small cell cancer treatment landscape for the first time in last 2 decades.

In the relapsed setting with prior exposure of platinum, immune therapy has not yet shown survival benefit and the FDA approval has been on the basis of response rates. Immune therapy has also not been compared to Topotecan (which does have a survival benefit). Cross trial comparison are fraught with inaccuracies however a real world experience does inform us about the significantly favorable toxicity profile of immune therapy compared to cytotoxic chemotherapy. A favorable toxicity profile with evidence of response could be convincing enough to use immune therapy instead of chemo in a relapsed SCLC setting. NCCN does not prefer immune therapy over chemotherapy.

In the chemotherapy naive setting, the evidence is clearer with a definite statistically significant survival benefit of adding Atezolizumab to Carboplatin and Etoposide. Addition of Atezolizumab did not add to the grade 3–4 adverse effects. Survival outcome in patient with varying plasma tumor mutations burden did not differ. It would be interesting to see if tumors with higher expression of PD L1 on tumor cells, or PDL1 expression on infiltrating immune cells had a better response and if solo Atezolizumab could be compared to chemotherapy in those patients.

Extensive small cell cancer is an aggressive disease with a poor prognosis and needs extensive research in identifying new targets for treatment. Targeting the check point inhibition axis is a step in the right direction.
